# Fulminant Course of Isolated Orbital Metastasis From Gastric Cancer: A Case Report

**DOI:** 10.7759/cureus.92117

**Published:** 2025-09-12

**Authors:** Leila Afani, Ilias Benchafai, Rhizlane Belbaraka

**Affiliations:** 1 Oncology, Centre Hospitalier Universitaire (CHU) Mohammed VI, Marrakech, MAR; 2 Otolaryngology-Head and Neck Surgery, Avicenna Military Hospital, Marrakech, MAR

**Keywords:** case report, gastric cancer, orbital metastasis, proptosis, signet-ring cell carcinoma

## Abstract

Orbital metastases are uncommon, with breast cancer being the most frequent primary tumor. In contrast, metastasis from gastric cancer to the orbit is exceedingly rare and often indicates advanced disease with a poor prognosis.

We report the case of a 43-year-old woman previously treated for localized signet-ring cell gastric adenocarcinoma with chemotherapy followed by total gastrectomy. One month after surgery, she developed acute right-sided proptosis, diplopia, and retro-orbital pain. Magnetic resonance imaging (MRI) revealed a right orbital mass encasing the globe, optic nerve, and extraocular muscles, consistent with a solitary orbital metastasis. Her clinical condition deteriorated rapidly, and she died within days of diagnosis. Histological confirmation could not be obtained due to her rapid decline.

Although rare, orbital metastasis may be the first and only manifestation of gastric cancer recurrence. Clinicians should maintain a high index of suspicion in any cancer patient presenting with new-onset ocular symptoms, as early diagnosis is critical for symptom management and prognostic assessment.

## Introduction

Orbital metastases are rare, accounting for approximately 1-13% of all orbital tumors and affecting 2-5% of patients with systemic malignancies [1]. The most frequent primary sites include breast (36%), melanoma (10%), and prostate (8.5%) cancers [2]. In contrast, orbital metastases originating from gastric cancer are exceptionally rare, with only a handful of cases documented in the literature [3].

These metastases may reveal an undiagnosed malignancy or signal recurrence in previously treated cancer. Diagnosis is based on clinical presentation, including proptosis, diplopia, or ocular pain, supported by imaging such as magnetic resonance imaging (MRI) or computed tomography (CT). Biopsy is recommended when imaging findings are inconclusive [4]. Management is often palliative, and prognosis remains poor.

We report a rare case of isolated orbital metastasis from signet-ring cell gastric carcinoma, where ocular symptoms were the first manifestation of systemic relapse, leading to rapid clinical deterioration and death.

## Case presentation

A 43-year-old woman presented with a six-month history of epigastric pain. Upper gastrointestinal endoscopy revealed a large ulcerated lesion in the gastric body. Histopathological analysis confirmed signet-ring cell adenocarcinoma. Initial staging by thoracoabdominal CT scan showed no evidence of metastatic disease. The patient had no significant past medical, surgical, or family history.

She was treated with four cycles of neoadjuvant chemotherapy, namely, docetaxel (50 mg/m²), oxaliplatin (85 mg/m²), leucovorin (200 mg/m²), and 5-fluorouracil (2600 mg/m²), administered as a 24-hour continuous infusion. Post-chemotherapy evaluation showed stable disease. She subsequently underwent total gastrectomy without postoperative complications.

**Figure 1:**
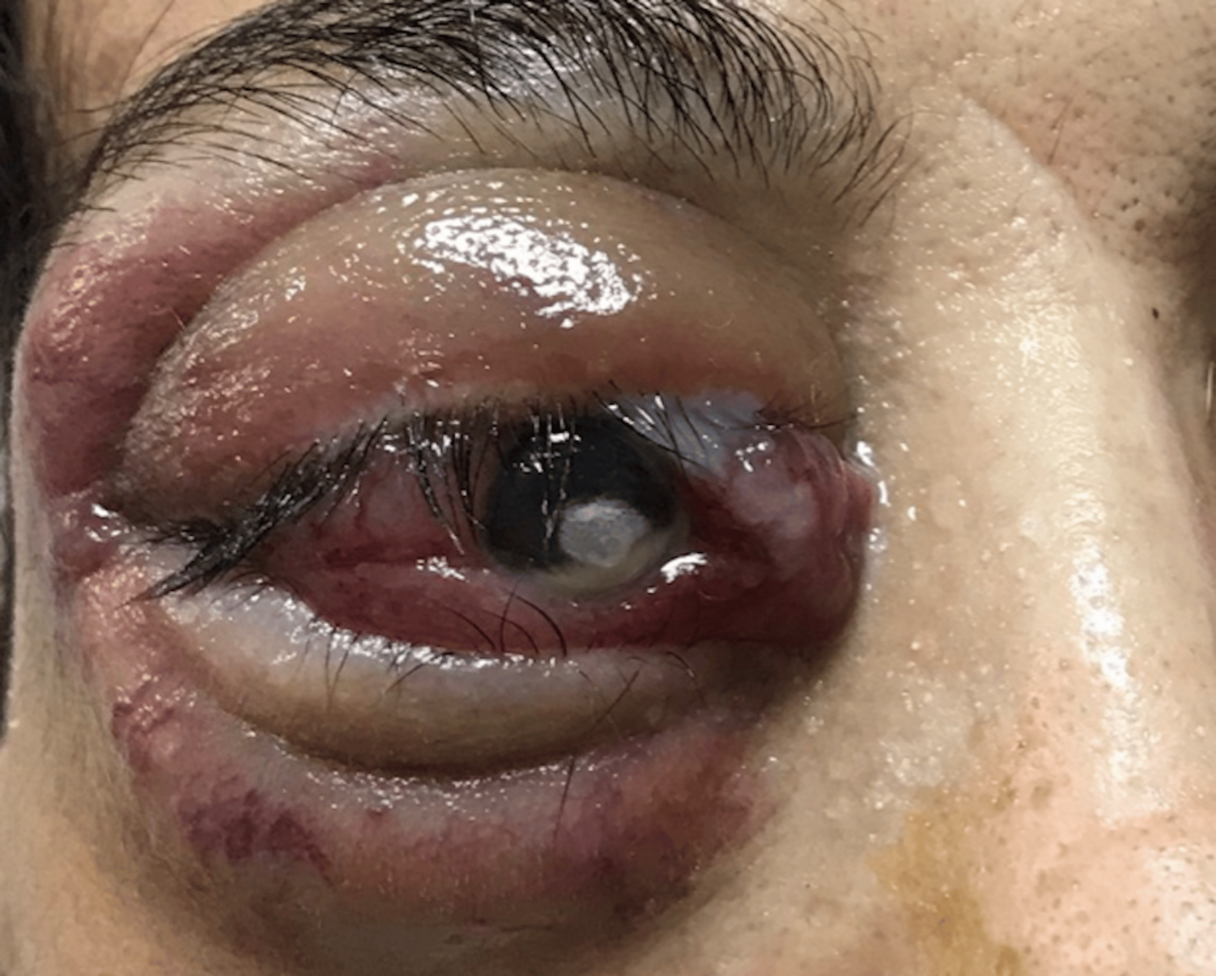
Photograph showing a severe proptosis of the right eye

One month post-surgery, the patient developed sudden-onset right-sided proptosis, diplopia, and retro-orbital pain (Figure 1). The ophthalmic examination showed unreliable measurement of right visual acuity, chemosis, eyelid edema, corneal opacification with a dependent level compatible with a probable hypopyon, and painful limitation of eye movements in all directions, suggestive of extraocular muscle involvement.

**Figure 2:**
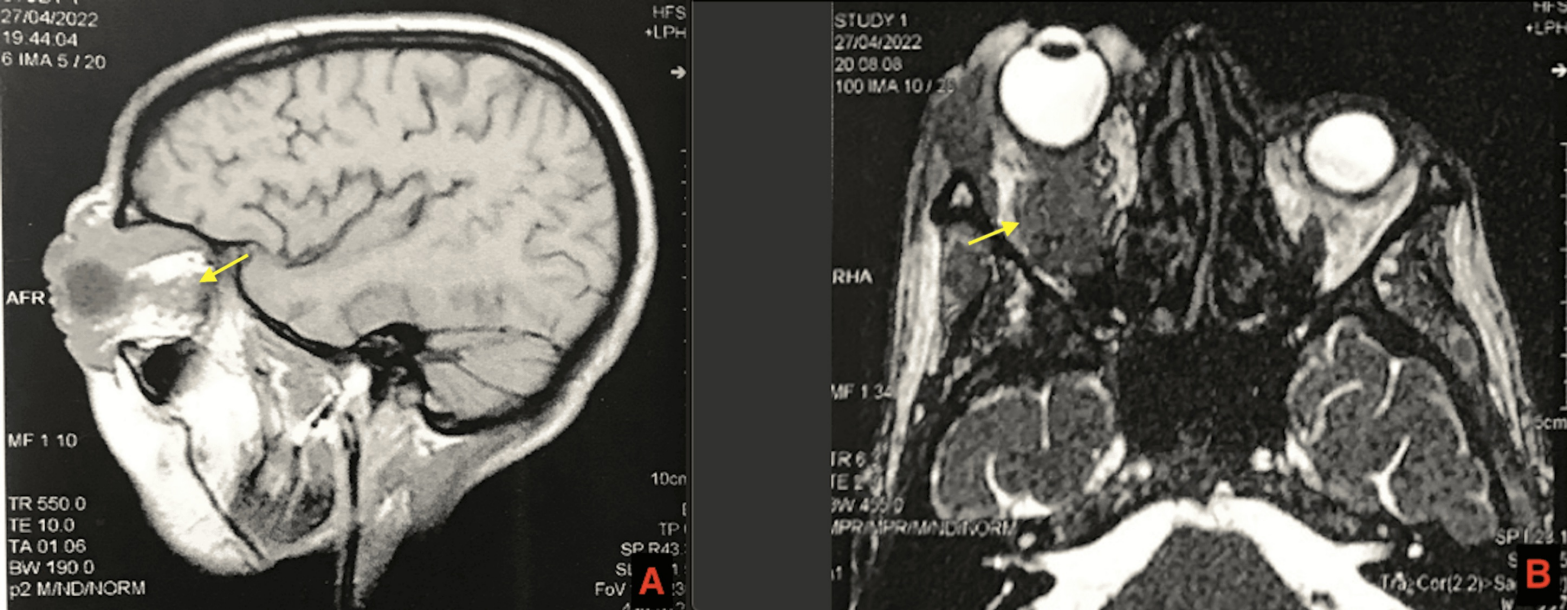
T2 axial (A) and T1 sagittal (B) slices showing a lesional process infiltrating the oculomotor muscles, the optic nerve, and the intra- and extra-orbital fat of the right orbit, with infiltration of the eyelid (yellow arrows)

Orbital CT showed extensive soft tissue infiltration of the right orbit. MRI confirmed a mass involving the globe, optic nerve, and extraocular muscles, consistent with grade III proptosis (Figure 2).

Additional lesions were noted in the left orbit and facial soft tissues. Follow-up CT of the chest, abdomen, and pelvis showed no systemic metastases. A biopsy was planned but could not be performed due to the patient's rapidly deteriorating condition. She died within a few days of diagnosis.

## Discussion

Gastric cancer remains a leading cause of cancer-related mortality worldwide. Common metastatic sites include the liver, peritoneum, and lymph nodes [5]. Orbital metastases are exceedingly rare, especially from a gastric origin. In a review by Amemiya et al., only 11 cases of orbital metastases from gastric cancer were reported over nearly a century [3].

Orbital metastasis may occur as an initial sign of undiagnosed cancer (19-25% of cases) or as a recurrence [6]. Typically unilateral, the orbit is affected in descending order: choroid, orbital tissues, iris, and ciliary body [7,8].

Common presenting symptoms include diplopia, vision changes, pain, and proptosis [9]. The average interval between gastric cancer diagnosis and orbital metastasis is approximately 25 months, and the mean survival after the onset of ocular symptoms is 3.2 months [3].

Diagnosis relies on imaging, with MRI being superior for soft tissue evaluation. Biopsy is warranted in isolated cases, in incongruent clinical/imaging findings, or when histology is needed for targeted therapy planning [4]. In our case, despite suggestive imaging findings, histological confirmation was precluded by the rapid clinical course.

Treatment of orbital metastases is typically palliative. Systemic chemotherapy, targeted agents, and immunotherapy may be considered based on tumor biology. Surgical resection is rarely indicated due to widespread disease. External beam radiation therapy (20-30 Gy) remains the mainstay of local palliation, with symptom control rates between 57% and 91% [10]. Advanced modalities such as proton therapy and stereotactic radiotherapy have shown promising local control in select cases [11,12].

Prognosis depends on the primary tumor type; breast cancer metastases to the orbit are associated with better survival compared to gastric cancer [13].

## Conclusions

Orbital metastases are a rare but significant manifestation of systemic malignancy. This case illustrates how such metastasis, diagnosed on clinical and radiological grounds given the infeasibility of biopsy, can serve as the first and only sign of gastric cancer recurrence. Clinicians should consider orbital metastases in any cancer patient presenting with new ocular symptoms, as timely diagnosis may aid in symptom management and guide treatment decisions, even if curative intent is not feasible.
